# Technological Transformation of Telco Operators towards Seamless IoT Edge-Cloud Continuum

**DOI:** 10.3390/s23021004

**Published:** 2023-01-15

**Authors:** Kasim Oztoprak, Yusuf Kursat Tuncel, Ismail Butun

**Affiliations:** 1 Department of Computer Engineering, Konya Food and Agriculture University, 42080 Konya, Turkey; 2 Department of Computer Engineering, KTH Royal Institute of Technology, SE-114 28 Stockholm, Sweden

**Keywords:** autonomous, distributed, OpenFlow, SDN, 5G, 6G, next-generation, IoT, edge-computing

## Abstract

This article investigates and discusses challenges in the telecommunication field from multiple perspectives, both academic and industry sides are catered for, surveying the main points of technological transformation toward edge-cloud continuum from the view of a telco operator to show the complete picture, including the evolution of cloud-native computing, Software-Defined Networking (SDN), and network automation platforms. The cultural shift in software development and management with DevOps enabled the development of significant technologies in the telecommunication world, including network equipment, application development, and system orchestration. The effect of the aforementioned cultural shift to the application area, especially from the IoT point of view, is investigated. The enormous change in service diversity and delivery capabilities to mass devices are also discussed. During the last two decades, desktop and server virtualization has played an active role in the Information Technology (IT) world. With the use of OpenFlow, SDN, and Network Functions Virtualization (NFV), the network revolution has got underway. The shift from monolithic application development and deployment to micro-services changed the whole picture. On the other hand, the data centers evolved in several generations where the control plane cannot cope with all the networks without an intelligent decision-making process, benefiting from the AI/ML techniques. AI also enables operators to forecast demand more accurately, anticipate network load, and adjust capacity and throughput automatically. Going one step further, zero-touch networking and service management (ZSM) is proposed to get high-level human intents to generate a low-level configuration for network elements with validated results, minimizing the ratio of faults caused by human intervention. Harmonizing all signs of progress in different communication technologies enabled the use of edge computing successfully. Low-powered (from both energy and processing perspectives) IoT networks have disrupted the customer and end-point demands within the sector, as such paved the path towards devising the edge computing concept, which finalized the whole picture of the edge-cloud continuum.

## 1. Introduction

Telecommunication operators (known as Telco) have been in a transformation. The data rates offered to mobile users are increasing exponentially. Today, 5G systems allow end-users to reach data rates of Gbps, which is tremendous and even higher than the current speeds of some fixed-line services.

During the last two decades, desktop and server virtualization has played an active role in the IT world, as well as for the Telcos who are still in the middle of the transformation, while all parties have experienced the effect of better resource utilization and ease of use. When OpenFlow [1,2], as the first building block of the SDN [3] was introduced, most of the telecommunication world was unaware of the birth of a revolution. The proposed solution was revisiting the early telephony networks with a clear separation of control and data planes. This is not surprising since almost all significant revolutions (e.g., ATM, MPLS, etc.) in the telecommunication world were revisiting original telephony networks’ ideas to simplify network management with better service quality to optimize the costs in the operations.

In the early days of SDN, multiple government-funded projects started to emulate large networks through the server infrastructure. Because of these efforts, controlling network devices is extended to control the physical and hypervisor-based virtual network devices. Successfully separating the control plane and data plane by defining a communication protocol, the need for intelligence appeared to perform the network-wide decisions and enforce the determined communication protocol to harmonize the networking. This demand is met by introducing SDN controllers.

The enormous progress in clarifying the picture in the control plane has sped up the data plane’s workings. The glue filling the evolution in networking and SDN was the network virtualization adapting itself to available heterogeneous hardware. In this aspect, NFV is introduced to utilize the specialized networking boxes. According to ETSI, SDN, and NFV are complementary to each other [4]. NFV allows the operators to replace the appliances for network functions such as firewalls, load balancers, and customer premises gateways by virtually delivering the services [4]. With the use of OpenFlow, SDN, and NFV, the journey to the network revolution has started. However, initially, it did not mean much to the Telcos because something was missing in the picture to motivate them to adapt or at least exhibit the benefits of these new solutions. The operator world was designed to have vertical SILOs within their management domains, which is not practical since they would have tens of thousands of more devices than they had in the past.

The operators’ missing part in the journey was harmonizing the policy management and orchestration systems with the capabilities of delivering new services and allocating resources dynamically upon the demand of the users and systems located in the SILOs. The change from SILO oriented legacy telecommunication services to the future of telecommunication systems, including subscriber-defined services with near real-time processing and very low delay and higher capacities without limitation to the access platform, has been critical.

**Figure 1:**
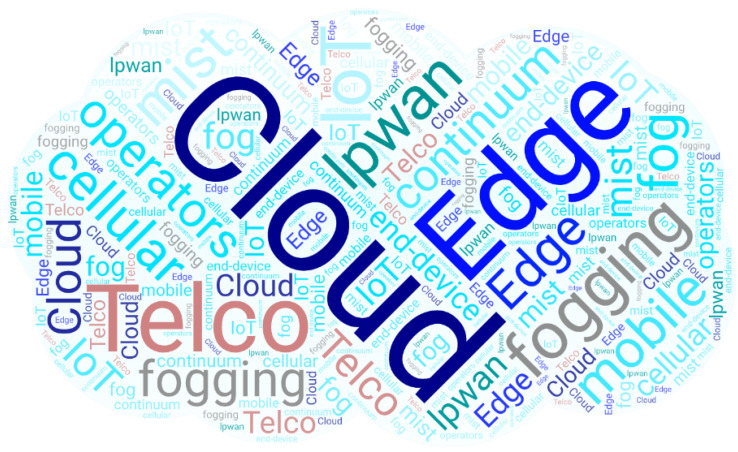
The word-cloud of the cloud-edge continuum concept.

As shown in Figure 1, the overall concept of the cloud-edge continuum consists of many ingredients, and it is a non-trivial task to present the overall concept in a single publication. There have been similar studies in this area; however, none of them presents an end-to-end picture defining all aspects of a Telco operator. We differ from the others by four critical elements:

1. Cover the whole picture for an operator from an insider view,
2. Present the progress in the control plane (SDN), data plane (NFV and others), and
3. Deal with the evolution of software development culture with the improvements in orchestration and automation platforms where the orchestration platform can be accepted as the brain with command-and-control capabilities in an operator.
4. Discussions on the positive effects and benefits of the cloud-edge continuum for IoT networks.

In this article, we present the fundamental changes that Telco operators have faced while their provided services are transforming voice-based to data and cloud-based computing services. We inspect the effect and impact of the edge-cloud continuum on key verticals such as Industrial IoT along with cyber-security, which has primary importance in industrial manufacturing, oil, and gas industries. Moreover, there is no doubt that seamless connectivity of the edge-devices, pervasive computing ability, and remote precision control of the machinery will be benefited from the provided services of the edge-cloud continuum [5].

Furthermore, we discuss the requirements of the new technological challenge so-called Edge-Cloud continuum, which is a promising yet challenging phase for Telco operators. The major prospects of this new service are thoroughly elaborated not only from the Telco operators’ domain but also from the partners’ and customers’ point of view. Thereafter, we examine the related work on SDN, NFV, SD-WAN, and similar advancements in technology which is required to assess the Edge-Cloud continuum, while giving an up-to-date comprehensive overview and delineating challenges and issues surrounding the studied schemes. Finally, we highlight open problems and potential future research directions.

## 2. Background

### 2.1. Virtual Machines, Containers, Dockers, and Kubernetes

One significant move triggering the change is the development of virtualization technologies. The networking side’s transformation is tightly coupled with the compute-side, especially in NFV.

The story started at the Massachusetts Institute of Technology for the MAC project [6], giving rise to the first time-sharing operating system (OS) to utilize all computer resources. In the early 90s, virtualization took the role of running a different operating system on top of a host OS to help the software compatibility. Although virtualization enabled users to run an OS and its applications on another hardware platform, later, it turned out to be a resource utilization technique to lower the costs. Afterward, VMWare became the flagship of computing resources’ virtualization, including servers and desktops.

Every virtual machine allocates separate memory and “compute” resources for its OS; thus, it should keep a copy of a network packet for itself while processing, which is one of the reasons for performance degradation. These performance problems and additional security concerns resulted in the development of containers.

Unlike Virtual Machines (VMs), containers are built on top of an operating system and managed by a container manager. Read-only libraries are shared among other containers. Container-based applications can be started in the order of seconds compared to a few minutes in VM-based applications. Containers utilized shared resources to a much higher degree than VMs, reducing the size of private components, and making them lightweight, resulting in higher granularity and higher performance than VMs.

Docker is the main player in container technology, allowing users to create, deploy, and run applications easily. In addition to more accessible packaging support, it also has a clustering tool to schedule and orchestrate clusters of containers across the VMs. Kubernetes is developed at Google to automate the deployment, scheduling, and scaling of containerized applications and support many containerization tools such as Docker, containerd, and any implementation of the Kubernetes CRI. Later it has been donated to Cloud-Native Compute Foundation as an open-source project. It is the de-facto standard in container orchestration with the capabilities of grouping containers into logical units allowing the systems to distribute containers into multiple physical nodes scaling up to enormous dimensions and load balancing. Using Kubernetes, the data center owners or operators can run multiple instances of an application and independent upgrades and versioning.

Although the change in the virtualization methods improves the performance of the systems, the change in virtualization is a revolution in:

1. Concentrating on the services rather than machines and structuring them as micro-services,
2. dynamically managing services by scaling, updating, and co-operating multiple versions as needed, and
3. grouping the containers to simplify management, enable load balancing, high availability, and deployment. This different approach comes from containers’ power in opening resource-efficient services quickly and retiring them similarly when the demand expires, maintaining resource and security isolation between services.

### 2.2. Evolution of Information Technologies and Related Environments

While the networks transform into software-defined, programmable, service-based infrastructures, the new paradigm should provide agility, scalability, and fully automated systems. Such phenomena in the world are always triggered by cultural changes in the way of doing business. One of the most significant moves in this paradigm is the cultural change in application development, deployment, maintenance and operation with a new mindset.

The movement from monolithic application development to micro-services changed the structure of all narratives. It started with the commencement of the cultural movement of application development named by Debois, as DevOps [4]. Although there are some formalized set of operational processes defining the workflow and relationship of service design, strategy, transition, operation, and continual service improvement like IT Infrastructure Library (ITIL), DevOps focuses on the productive collaboration of software developers and IT operation personnel by changing attitudes, processes and team interactions. Unlike ITIL, there is no clear delineation in DevOps. It uses agile software development methodologies and applies them to automate all software life-cycle steps from development to deployment for operation. DevOps eased the burdens of traditional SILOs and brought agility into the whole application and operation life-cycle.

### 2.3. Cloud-Native, Edge Computing, and Microservices

The cultural shift with DevOps brought agility, effective use of microservices architectures, and continuous integration and development (CI/CD) workflows. Containers became the most suitable implementation platform for microservices-based, agile applications. This total change enhanced the applications to run in a cloud environment smoothly. Recently, “cloud-native” is a popular term used to define the applications and services capable of running in cloud environments. While cloud services focus on overall user experience and the companies’ internal IT compliance, cloud-native services focus on delivering the massive scale of applications due to their small and stateless nature, which increases mobility and scalability. Cloud-native services span across data centers, Edge, and user devices. A Cloud-native mindset is a key to leveraging “compute at the Edge” [7]. The architectures leverage the accountability for computing and communication while bringing intelligence to the Edge.

The customers’ changing demands helped shift the focus of service providers from traditional virtualization-based data centers to simple, flexible, microservice-based cloud-native services using Linux containers [7]. This change allowed service providers to speed up the rapid development and deployment of new services to scale up and down upon changing demands and traffic patterns.

### 2.4. Data Centers

Data centers evolved over several generations. In the first generation, they aimed to optimize the CAPEX mainly in hardware by using virtualization. The second generation of data centers, currently in use, uses public clouds, which frees enterprises to set up their physical systems. The business logic is still the same and manages all the systems remotely. The evolution from the first generation to the second generation can be summarized as changing from hardware to software-based data centers.

The current generation is serverless computing, which is in its early steps of deployment and production. In this generation, one does not deal with today’s daily tasks such as OS configuration, load balancing, or patching systems, but only needs to deal with the computing capacity or services. The customers’ objectives will be the quality of services throughput or latency of the applications or functions rather than several VMs or the amount of storage.

In the future, with the mass deployment of 5G, the base stations will become the extension of the data centers at the edge. Extending data centers towards the edge will allow operators to deliver fast-lightweight computing services with minimal delay for the applications that need such services where more than 50% of the data could be stored.

### 2.5. Programming the Hardware in Run-Time

As stated previously, the telecommunication world is in great transformation. The most important aspect of this transformation is to switch from old hardware-dependent, vertical architecture to software-defined architecture. In this architecture, there are a series of improvements compared with the current products. Although the use of NFV was a key improvement in the data plane with improved flexibility, Protocol Independent Switch Architecture (PISA) is one of the key elements with accelerated performance and intelligent processing ability in the data plane during this change. The change in the architecture affects all stakeholders in a telecommunication operator infrastructure including applications. Legacy Applications written for legacy hardware are transformed into Software-defined architecture [8].

Independent from the Software Defined Architectures, application identification and control became critical in the last decade. It perched itself at the center of cybersecurity, accounting, quality of service management and similar services. One of the most important problems incurred by application identification is the resource-hungry behavior of the process. Next-Generation Firewalls (NGFW) and Deep Packet Inspection (DPI) systems are two of the most popular usage area of application identification and control. DPI, as the name implies, inspects every packet that is running through the network deeply, and tries to classify it under a human-readable name. It not only relies on packet metadata and header but also packet payload, hence the name “Deep”.

With the emergence of SDN architecture, DPI vendors or similar packet processing solution providers switched from hardware to software-based solutions. As they switch from hardware-dependent architecture to SDN-based architecture, they lack the proper scalability to match the actual line speed of the switches. While the capacities of the data backbone increase, the systems depending on application identification became the bottleneck of the infrastructure.

As explained before, the network applications become Virtual Network Functions (VNF). As the demand increases, the telecom operators will need application identification systems like NGFWs and DPI systems running with the speeds in the order of Tbps of traffic classification in real-time and such as in a single instance of DPI. The performance gain arises from the fact that the classification operation starts at the switch-level code data plane and continues in the user plane.

## 3. Telco Operators’ Point of View

### 3.1. Catching the Changing Demands of a Telco Operator

Although the research and adaptation are spreading among the operators, most decision-makers and adapters are confused about what SDN is and what it will bring. There are also promotional efforts by some vendors, trying to position SDN as a blue pill to cure all the existing problems of the operators. Indeed, it would even increase the complexity of the issues without proper planning.

Before discussing SDN, NFV, and their impact, it is better to define the status quo and future expectations of an operator.

**Figure 2:**
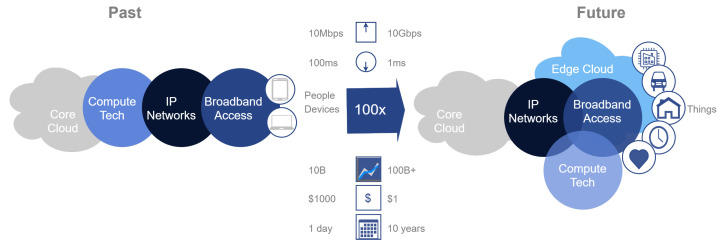
The change in the capacity demand, latency, and services in the telecommunication world [8].

As depicted in Figure 2 in a traditional operation, the operator’s principal assets are the transmission infrastructure bringing the connectivity between the core cloud and the broadband access for the subscribers. Typically, the operators are hosting their computing infrastructure, mainly for hosting Operation Support Systems /Business Support Systems (OSS/BSS), and some services through data centers in the core cloud. The subscribers are accessing communication services through broadband access, while an IP communication network forms the backbone. The design is simple, and the only purpose of broadband access is to connect the subscribers to the services. The computing technologies provided by a conventional operator are aggregated into a few data centers.

In the new era of computing, the architecture of the operators will change slightly. The technology will shift from the core data centers toward the Edge and form a new edge cloud through a new generation mobile access cloud besides the computing power. While the core cloud, IP backbone, and access will live in their position, their structure will transform. First, the data centers will have extensions with enough computing power at the Edge delivering near real-time processing power, especially for systems with the need for low latency like autonomous devices, IoT, and caching the traffic intelligently to reduce the traffic load through the network. This also triggers the profile of the users accessing the services heavily from the people to the things. The new architecture conforms to the telecommunication world’s catchword: distribute when you can, centralize when you must.

Leading operators in the telecommunication world started to adopt SDN into their network to build a network infrastructure that will optimize costs and spin out new services faster than the current situation for their customers. NFV is the complementary technology in creating the target telecommunication architectures for SDN-NFV transformation. The impact of SDN and NFV technology on the carrier network is heavily observed in provisioning, capacity planning, key performance indicators (KPIs), infrastructure service, and security.

The change in the telecommunication systems is not limited to the infrastructure’s architecture but mainly focuses on the way of approaching subscribers as well as the services. The systems are changing in favor of customer-oriented and service-based systems rather than system-oriented subscriptions. This change in subscribers’ approach ultimately needs end-to-end automation from service requests to delivery, including provisioning, maintenance, and service closure. The adaptation of SDN and NFV is transforming the networking and changing the culture and roles of people in the IT chain, like what DevOps brought to the software development and operation life cycle. Telecommunication networks are transforming to become a new infrastructure paradigm than merely extending the Cloud by considering the evolving “Edge” demands. This shift is not limited to but includes the adaptation of edge computing, which becomes mandatory for 5G and IoT applications in real-time.

### 3.2. Expectations of the Telco Operators

In the past, most of the operators were only concentrating on the availability of the services. The box-oriented approach in telecommunication infrastructure resulted from this basic approach favoring simplicity. It was easier to implement a single application without considering integrating it into other systems. Availability is still the first expectation from a system to guarantee stability and design with security concerns. An insecure platform is unavailable since someone else shares its control with immediate access to the systems. The redesigned systems are trying to disaggregate the systems’ parts to reduce the complexity of the systems which will also increase the availability. Disaggregation will be the key to improving availability.

Although availability is crucial for the services’ existence, there was limited manageability support for the box-oriented vertical systems. Manageability brings visibility to systems where performance does not matter. Automation is the part or mutated form of management favoring agility while designing new services and quickly delivering service to a customer. Automation is crucial to reduce the flexibility in the availability by enforcing the systems to comply with some standards or way of doing their tasks, which would lead them to lose some functionalities or performance.

With the burden of continuous evolution, the future of the integrated telecommunication world covering cloud, edge computing, and data centers, including cloud-native applications, services, and portability of them, will be built by using open-source tools tightly integrating and orchestrating across containers. This evolution in open-source technologies shifting all communication systems into the next phase also requires some new arrangements on compliance (regulations) and security protocols with being fully audit-able to ensure providing acceptable service level and supporting common identity and access management, policy management, and a full range of service portability [9].

### 3.3. Resulting Changes for Telco Operators

The operators are developing their products to survive within the competitive environment in a “saturated market”. This allows them to deliver any feature, any time, reducing the service time of a new service or a new feature in a current product to almost zero while having it with incredibly cheaper costs. The operators are becoming part of open-source initiatives to have flexible and less expensive systems and assuring security across the network.

In both fixed and wireless open-source projects, the intelligence embedded in the legacy access equipment is taken out by disaggregation, resulting in simple hardware with sophisticated software. This idea is supported further by transforming monolithic eNB into disaggregated control and data units with network slicing support. Disaggregation is the key to this journey, which requires a redesign of network hardware and software [10]. Disaggregation allows researchers to work with a smaller subset of a problem at a time, leading to a speed-up in innovation while helping the technology users optimize the resources they use (such as reducing the hardware usage). As a result, the network equipment evolved from a legacy black-box to disaggregated programmable equipment with control-data plane separation. One such example is Cloud Radio Access Network (RAN), which is proposed to create a programmable radio environment. Its main aim is to reach optimized converged networks. The total target in the cloud RAN is to accommodate the expectation of future services. Several initiatives define the standards of cloud RAN, such as O-RAN attracts the most among the others.

Disaggregation triggered the consolidation of the systems. Telco operators having a mobile and wire-line services will consolidate the control planes in a simple system. Simplification of the user plane will allow operators to manage users as a single entity regardless of the subscription diversity to the services. The convergence in the control plane will let the operators define end-to-end network slicing and even bringing a computing resource usage service as a combination of cloud and edge computing resources.

Several factors are forcing the operators to join this journey. Although the cost of serving the customers seems to be the priority, it would not be fair to put it in the first place. A notable reason is the time-to-market. Once the operators use the SDN, it will help them deliver a new feature to all users almost within a week compared to 18 months with the legacy systems delivered by vendors. Hence, it is evident that using open-source brings far faster deployment, updates, and innovations.

To succeed in this journey, success should be demonstrated publicly. Hence, network operators publicly stated that they are transforming their networks into a platform for innovative services and building the “network as a platform” with SDN/NFV/Cloud with dis-aggregation, open-source, white boxes to reduce Capex and Opex significantly [10]. More interestingly, those changes recall the catchword: “Network is the computer”. This now applies to our case as: “the operators’ infrastructure is the computer.”

## 4. Developments in the Edge Continuum: Automation, Security, SD-WAN, Blockchain, and IoT

### 4.1. Leveraging the Automation

The final part of completing the picture in a carrier-grade network is the coordinator working closely with the operation of the applications and services that generally run on the network and the underlying infrastructure. The industry uses the term orchestrator to coordinate and manage all network and compute elements needed to deliver a virtualized network service, including provisioning.

Several open-source products in NFV management and orchestration (MANO) solutions are proposed based on the released ETSI standards. The most promising automation software among the orchestrators, Open Network Automation Platform (ONAP) by Linux Foundation Networking, is almost becoming the de facto standard for the real brain of the whole infrastructure for an operator. Although OSM itself is owned by ETSI, the maintainer of the standard, it lacks critical features such as Kubernetes support, PNF integration, edge automation, real-time analytics, network slicing, data modeling, homing, scaling, and network optimization, as shown by a recent study [11].

**Figure 3:**
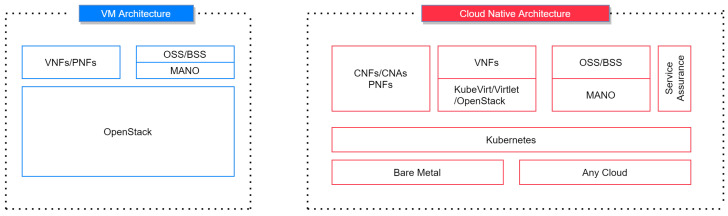
VM Architecture vs. Cloud-Native Functions.

To leverage the automation, Cloud-Native Architecture is critical. Figure 3 shows a brief difference between VM Architecture and Cloud-Native Architecture, as demonstrated by Kapadia, A. [12]. The demonstrations aim to show how easy to onboard 5G core and Next-Generation Firewall with Cloud-Native Network Functions using ONAP, OPNFV, and Kubernetes, along with the working demonstrations and end-to-end testing in a lab environment. As indicated in Figure 3, in current Telco VM Architecture, the main player is OpenStack. On the top of OpenStack, VNF’s/PNF’s are positioned in order to eliminate vendor-locking. OSS/BSS and Management and orchestration (MANO) are placed at this level. This architecture is evolving into Cloud Native Architecture, replacing OpenStack with bare-metal deployment with cloud-agnostic methods, putting Kubernetes/Docker over the top of it for managing micro-containers, pushing OpenStack to an upper level, positioning the rest of the classical Telco Management components at this level.

Going one step further, zero-touch networking and service management (ZSM) is proposed to get high-level human intents to generate low-level configuration generation for low-level devices and controllers with validated results. The main target of ZSM is to minimize the ratio of faults caused during a human intervention.

### 4.2. Next-Generation Security Services in Telecommunication Networks

Modern problems require modern solutions. As the SDN/NFV enabled networks and operators emerge, customers’ cybersecurity services will shortly be shaped differently.

Providing security-as-a-service could be one of the ultimate goals of telecommunication networks. As the processing power of network devices increases, security services are moving toward the Edge. In a recent study with an attractive title, “Towards security-as-a-service in multi-access edge” [13], authors propose a data-centric SECurity-as-a-Service (SECaaS) framework for elastic deployment and provisioning of security services at the Multi-Access Edge Computing infrastructure.” Motivated by the rapid growth of the Industrial Internet of Things (IoT), autonomous driving, and smart home applications, and the shortcomings of security measures taken at the core network to secure the services, authors suggest a novel security architecture that should be offered at the near edge of the network for tenants with different requirements by using the Named Data Networks (NDN) architecture.

To offer security services, the underlying system architecture should be robust and secure as much as possible. In a recent survey on SDN-NFV security [14], authors conclude that at least three central issues and potential research areas are:

1. The performance impact of enhancing security in SDN-NFV networks,
2. the importance of detecting abnormal behavior within the layers by monitoring,
3. the security issues related to OpenStack.

Another recent research [15] conducted about the security algorithms that are used on cloud platforms draws attention to important points. The new analytical model presented in this research is designed for multiple-cloud platforms in consumer data transmission and aims to analyze and address issues related to data errors. The model offers data synchronization and latency reduction for both uplink and downlink data within each cloud database. It also investigates risks associated with using a single cloud platform for large data operations and proposes energy-efficient solutions. A control data system is also implemented to connect data across various tasks that the cloud system must perform. The multiple cloud systems are more efficient when used on a schedule, so the proposed solution aims to reduce data delays during transmission. The use of the Improved Blowfish Algorithm (IBFA) helps to reduce the risk of data collision by allocating sufficient resources for the entire cloud database, resulting in a more adaptable wireless transportation operation.

### 4.3. What Is Changing on the Edge Side?

The term “edge computing” was first used by Akamai Technologies in 2002, which obtained a patent in 2004 [16]. In their context, edge computing was a particular methodology to deliver Java-based application content responsively to the web-browsers for a better user experience.

A more recent and relevant definition from the chair of the Mobile Edge Computing group of ETSI, Reznik, is “anything that’s not a data center cloud” [17].

The revolutionary change at the core and access triggered SD-WAN’s evolution to fulfill the customer’s needs. The operators currently deliver simple L2 or L3 pipes as a VPN service with minimal traffic engineering support, mainly through their MPLS networks. The Edge in the future should support application-centric slicing and traffic engineering besides the current L2/L3 pipes. Another new improvement on the WAN side is Service Function Chaining (SFC). SD-WAN solutions bring full flexibility to the customer’s aggregating network functions from different vendors into a single box and enable ease of access to the cloud.

Actively using SDN and NFV allowed operators to define a workflow for any kind of customer data flow through SFC’s help. The ability to define customized paths for any data flow reduces the need for resources since only the prescribed flows pass through any network function contrary to the current deployments. All data flows pass through all functions residing in scalability and high resource consumption problems. SASE is becoming the dominating security-cloud services for the customers leveraged by SD-WAN.

SD-WAN [18] is one of the enablers of hybrid cloud ecosystems combining on-premise and cloud-based applications. SD-WAN solutions bring full flexibility to the customers’ aggregating network functions from different vendors into a single box and enable ease of access to the cloud. SD-WAN eliminates the need for MPLS between a central office and branch offices by using software-based techniques to reduce the need for high-speed connections, providing built-in packet-level security, de-duplication, and data caching.

The evolution of telecommunication technologies, especially shifting from hardware-based systems to Software Defined Networking enabled entrepreneurs to design new systems and services. Helium [19] is a great example of such a case. A new service aggregating/connecting base stations/network nodes to deliver network access service to LoRa users. The node owners/managers are motivated to earn crypto tokens named Helium upon providing liveliness and access for the users. The system is designed to work hand to hand with a Crypto Economics system. This is a good example of SDN enabled IoT service based on LoRa motivating the node managers to earn tokens. Solving the economics in such a way reduced the cost of managing the whole operator. Later, Helium started to build a 5G network which has some complications due to regulations, however, it is growing very fast.

Multi-access Edge Computing (MEC) is a network architecture concept that allows for the deployment of computing and communication resources at the edge of a telecom network, in close proximity to end users. The main idea behind MEC is to bring the computing power and storage capacity closer to the users, so that data can be processed and stored locally, instead of having to be transmitted to a distant data center. This can help to reduce latency and improve the performance of applications and services that rely on real-time data processing, such as virtual and augmented reality, autonomous vehicles, and the Internet of Things (IoT).

MEC technology can be used in a wide range of use cases, including:

1. Improving the performance of latency-sensitive applications: MEC allows for data to be processed and stored closer to the users, which can help to reduce latency and improve the performance of applications that require real-time data processing, such as virtual and augmented reality, remote surgery, and autonomous vehicles.
2. Offloading traffic from the core network: MEC can help to offload traffic from the core network by allowing data to be processed and stored locally at the edge of the network. This can help to reduce congestion and improve the overall performance of the network.
3. Enabling new business models and revenue streams: MEC can enable new business models and revenue streams by allowing telecom operators to offer value-added services, such as content delivery, data analytics, and IoT applications, at the edge of the network.
4. Enhancing security: MEC can help to enhance security by allowing data to be processed and stored locally, rather than being transmitted over the network to a distant data center. This can help to reduce the risk of data breaches and other security threats.

In short, MEC can help operators in the following areas:

- Reduced latency: MEC can help to reduce latency by allowing data to be processed and stored closer to the end users.
- improved performance: MEC can improve the performance of latency-sensitive applications by reducing the distance that data has to travel.
- Increased efficiency: MEC can help to offload traffic from the core network, which can improve the overall efficiency of the network.
- New business opportunities: MEC can enable new business models and revenue streams by allowing telecom operators to offer value-added services at the edge of the network.

On the other hand, it’s not straightforward to deploy MEC, there are a few issues such as:

- Cost: Deploying MEC infrastructure can be expensive, particularly for telecom operators that need to build new edge data centers or upgrade existing ones.
- Complexity: MEC can introduce additional complexity to the network architecture, which can make it more difficult to manage and maintain.
- Interoperability: MEC systems from different vendors may not be compatible with each other, which can create interoperability issues.
- Security: MEC systems may need to be secured against cyber threats, which can be a challenge.

### 4.4. Blockchain and Crypto-Based Services: Game Changers or Hype?

**Figure 4:**
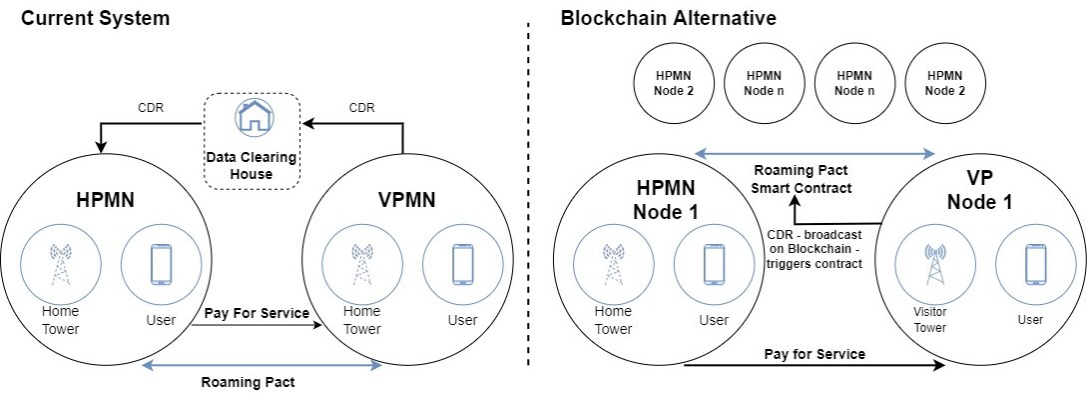
Blockchain for Telco Operators (inspired from [27]).

Blockchain in telecommunication networks gain high popularity with the hype. The research heavily focuses on the usage of blockchain in marketplaces [20], network sharing [21], infrastructure sharing [19,22], bandwidth sharing [23] and usage of AI in IoT [24]. Summarizing the benefits of blockchain in telecommunications, we see the following merits: decentralized, tamper-resistant, traceable, anonymity-preserving and transparent. Blockchain can help telco operators by enhancing privacy, security, and trust for future 6G networks [25]. Even 5G’s communication architecture ensures the transport of enormous volumes of data and information. With blockchain, terms and agreements may be negotiated instantly as digital smart contracts between access nodes, networks, and subscribers. Intelligent systems can autonomously authenticate user access, detect dangers, and eliminate malicious access from the internet. Currently, Communication Service Providers (CSPs) that deploy 5G plan to form alliances and use blockchain technology to encourage resource sharing and inter-operator network settlement [26]. According to Deloitte [27], blockchain can help CSPs in the following areas: Fraud detection, identity management, 5G Enabling and IoT connectivity. Blockchain may potentially ease or eliminate roaming frauds that put the burden of paying for overused services as shown in Figure 4, sim cloning, identity theft; provide new opportunities in identity-as-a-service applications, a new type o fix-mobile convergence, where WiFi‘s become part of the communication infrastructure and get paid back as a communication node; autonomous low powered wide-area network for IoT/M2M infrastructure sharing.

Sharing network resources as a source of revenue has been realized in 3 examples we have come across recently: Helium [19], LoRaCoin [22], TelCash [23]. Helium has been a commercial success for a shared infrastructure to build an intra-national LoRa (Long Range Wide Area) network. In this model, approved LoRa Gateways are installed by the user and connected to Helium network, and the users generate revenue as much as the gateway is utilized by LoRa devices and gateways. LoRaCoin uses a similar approach as Helium, but it proposes an open-standard instead of proprietary code and techniques used by Helium. TelCash proposes network bandwidth share among the mobile service users to generate revenue based on their excessive bandwidth to share with the other users in need, like in roaming cases, who crave network access in limited conditions.

Based on the current trends, we foresee that the hype has still not reached its peak value, and the research on blockchain in the telecommunications field will continue to grow. We will observe the results as the mass deployment of 5G networks gain momentum throughout the world.

### 4.5. IoT as a Beneficiary of Cloud-Edge Continuum

The rise of the IoT networks brings many breakthroughs with it, such as remote sensing and actuation, automation, observation, etc. which helps in many application areas such as smart cities, farms, buildings, etc. These comforting applications however bring their technological challenges, especially toward the IoT end-devices, such as high-processing power and memory-requiring computing operations. As such, edge-computing, and therefore, all advantages provided by the telco (4G/5G/6G) supported Cloud-Edge continuum will benefit the IoT networks in their high processing and memory-demanding tasks.

It’s important to note that the main goal of Telcos’ deploying 6G wireless communication networks is to use the Internet of Things (IoT) to create fully intelligent and autonomous systems. In a recent survey in 6G IoT [28], the authors look at the potential benefits of using 6G technology in IoT networks and applications. They describe several key technologies that are expected to be used in 6G, including edge intelligence, reconfigurable intelligent surfaces, and space-air-ground-underwater communications. The authors also discuss how 6G could be used in a variety of IoT applications, including healthcare, transportation, and manufacturing. Finally, they identify some of the challenges and opportunities for further research in this area. We anticipate that 6G will significantly improve internet of things (IoT) networks and enhance the quality of service and user experience in future applications.

## 5. Discussions and Conclusions

**Figure 5:**
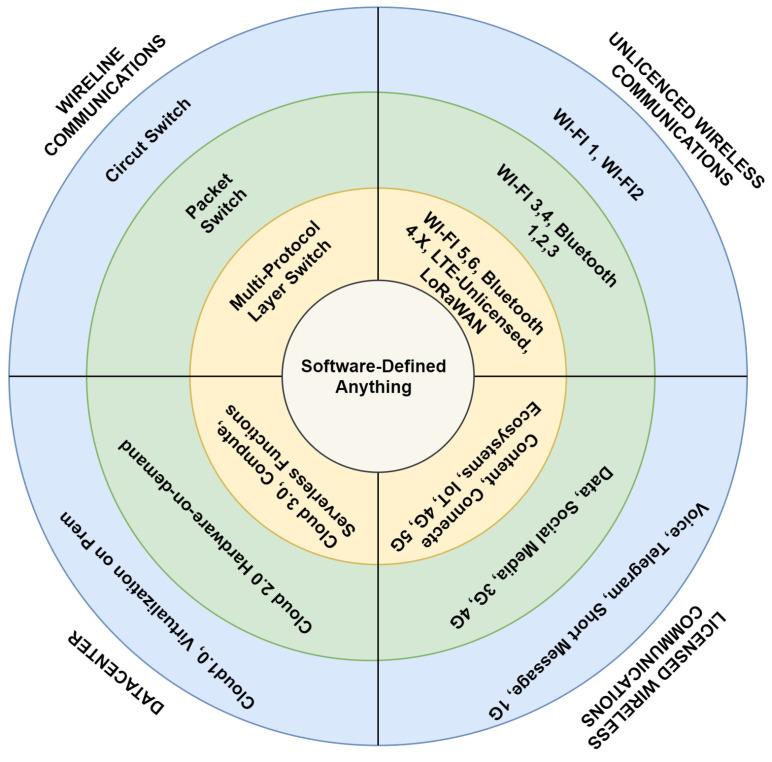
Mind map for the Transformation of the Networks [8].

The transformation in networking moves from legacy concepts into SDN-NFV based flexible, dynamic, agile, and more straightforward operation. As the orchestrators bring dynamic resource management and service provisioning, yet comes the zero-touch networking. The mind map in Figure 5 gives a brief explanation of the continuous transformation of networks toward “Software-Defined Anything”.

5G is a distinct business enabler for telco operators with different economic dimensions that brings the long-awaited ”programmable multi-access to the edge.” This is an evolution in access technologies where the operators touch the customers through the Edge. Although 5G is on the rise in parts of the world, it is crucial to start the aforementioned services through any band (4G) regardless of the spectrum, which will enable the technology developers and operators to test their service while allowing the subscribers to see what is being promised in a nutshell.

Due to the operators’ constantly falling incomes and their vast amount of investment in infrastructure, local regulators will allow or even encourage the aggregation of the common resources for active sharing among multiple operators, which will be a key enabler in the global services allowing the era of global Telcos, delivering worldwide services with the help of SDN-based technologies that will allow multi-national operators to access national infrastructures more easily. Helium is a low-cost alternative to conventional telco operator infrastructure. It transfers the cost of investment and management as well as provides network connectivity to the volunteer network node owners. In the future, similar low-cost operators will rise with competitive prices due to almost zero CAPEX and OPEX. The glue enabling such service is the Orchestrator.

In the past, the decision to enter the telecommunication market for a startup or a big vendor was based on their strong abilities in hardware design and delivery capabilities. However, this type of competitive advantage will lose its importance as the transformation steps up. With the SDN, the competition will be determined by the effectiveness of communication protocols and agility to deliver the software.

These changes result from the evolution of software and intelligent systems technologies. During the early times, we were talking about “Information Technologies (IT)”. Later, this turned out to be “Information and Communication Technologies (ICT)”. The future may bring “Information, Communication and Data Technologies (ICDT)”. This convergence of the technologies shifted the focus of operators to data technologies that use AI and ML techniques effectively to increase the monetization from data processing such as subscriber data, capacity management, planning, or churn management. AI has a big potential to help telco operators attain their strategic and financial goals. That is why they need to accelerate their AI transformation without any delay.

This will allow the stakeholders to use Telemetry data coming through anywhere to derive intelligent results such as assisting the network or profiling the customers/devices for monetization purposes [29]. The ambition toward AI/ML-based solutions for complex problems such as 5G management is not always easy to achieve. According to Benzaid and Taleb [30], although AI is seen as a critical factor for lowering operational costs and reducing the risk of human error, potential limitations and risks exist in using AI techniques. The authors summarize these limitations in 4 topics: (i) Lack of Datasets and Labeling, (ii) AI Model Interpretability, (iii) Training Time and Inference Accuracy, and (iv) Computation Complexity.

Similarly, the massive amount of telemetry data collected from network devices requires novel approaches and techniques to develop full-fledged, usable AI models. One of the most recent studies in this area [31], aims to solve the autonomous placement of Virtual Network Functions (VNFs) in 5G networks. Instead of using Supervised Learning (SL) models, the researchers used a particular form of Adaptive Reinforcement Learning. They achieved prediction accuracy performance gain by 40–45%, and overall VNF placement efficiency over against other SL benchmarks in 23 scenarios out of 27. This particular technique decouples the AI model from the training nodes, whereas other SL models are tightly bonded to the training nodes.

The automation platform will act as the operating system (OS) of a computer, while the management system of the network elements will act like a device driver communicating with the OS through their NBIs. ZSM is the beginning of autonomous telecommunication systems, which is not a dream anymore in the existence of SDN-based AI-driven automated-orchestrated infrastructures. With the increasing number of services to be opened for each connection, and the number of telemetry data collected for a device/service, automation/orchestration is a must for the future of telecommunication systems.

The recent past has shown that IoT networks require powerful and energy-supplied edge-components to delegate tedious processing-power and energy-consuming tasks [32]. Edge computing concept comes into help to ease these enormous tasks by employing fully-equipped edge-components nearby on-site deployments. However, edge computing also brings new challenges such as large bandwidth and high speed requiring communication ways, especially between the edge components and the back-haul (servers, etc.) of the networks. At this point, telco operators can help to tackle this problem by providing means of high-speed and reliable cellular communications, such as 4G/5G/6G, etc. As well as reducing the cost of communication and energy expenditures.

Edge computing is one of the biggest differentiators for telco operators when compared to cloud operators or Over-The-Tops (OTT). It is built around the point that they physically touch their customers while no one else can. This makes the Edge a unique differentiator for telco operators as well as a critical component of their 5G and IoT strategy in the next area of innovation for building new business opportunities. Even further, Wi-Fi and 6G will converge into a single service platform extending the fixed-mobile convergence concept to provide better security with end-user management capability.

The shift from centralized service delivery to edge computing is also a potential solution to high network cost and computing power requirements incurred from delivering add-on services such as deep packet processing services. In the future services to measure the capacity of a physical or overlay network for IoT systems will be a module to automation systems to build new services accordingly.

## Data Availability

Not applicable.

## Abbreviations

BSS	Business Support Systems
Capex	Capital expenditures
CI/CD	Continuous Integration and Development
CSP	Communication Service Providers
DPI	Deep Packet Inspection
ICT	Information and Communication Technologies
ICDT	Information Communication and Data Technologies
IT	Information Technology
ITIL	IT Infrastructure Library
KPIs	Key Performance Indicators
MANO	MANagement and Orchestration
NGFW	Next-Generation Firewalls
NDN	Named Data Networks
NFV	Network Functions Virtualization
ONAP	Open Network Automation Platform
Opex	Operating expenses
OS	Operating System
OSS	Operation Support Systems
OTT	Over-The-Tops
RAN	Radio Access Network
SECaaS	SECurity-as-a-Service
SFC	Service Function Chaining
SDN	Software-Defined Networking
SL	Supervised Learning
VM	Virtual Machine
ZSM	Zero-touch networking and Service Management
